# Transcriptome Analysis and Autophagy Investigation of LoVo Cells Stimulated with Exosomes Derived from *T. asiatica* Adult Worms

**DOI:** 10.3390/microorganisms9050994

**Published:** 2021-05-05

**Authors:** Panhong Liang, Yanping Li, Li Mao, Tingli Liu, Shaohua Zhang, Muhammad Ehsan, Liqun Wang, Aimin Guo, Guoliang Chen, Xuenong Luo

**Affiliations:** 1 State Key Laboratory of Veterinary Etiological Biology, Key Laboratory of Veterinary Parasitology of Gansu Province, Lanzhou Veterinary Research Institute, CAAS, Lanzhou 730046, China; liangpanhong628@163.com (P.L.); lyyp223@163.com (Y.L.); mao-li@live.cn (L.M.); ltl1114@163.com (T.L.); zhangshaohua01@caas.cn (S.Z.); mehsan124@gmail.com (M.E.); WLQ1282690114@163.com (L.W.); guoaimin@caas.cn (A.G.); glchen2019@163.com (G.C.); 2 Key Laboratory of Veterinary Biological Engineering and Technology, Institute of Veterinary Medicine, Jiangsu Academy of Agricultural Sciences, Ministry of Agriculture, Nanjing 210014, China; 3 Jiangsu Co-Innovation Center for the Prevention and Control of Important Animal Infectious Disease and Zoonoses, Yangzhou University, Yangzhou 225009, China

**Keywords:** *Taenia asiatica*, exosome, RNA-seq, autophagy, interaction

## Abstract

*Taenia asiatica* is a zoonotic parasite found in the human intestine and pig liver that evolved various strategies to survive the host’s defenses. Exosomes are membranous vesicles released by cells and are an important vehicle in parasite-host interactions. However, no literature exists on the specific infection mechanisms of *T. asiatica* against the host defense response, and further research is required to understand the parasite-host interaction. In this study, we investigated the host’s differentially expressed genes (DEGs) while stimulating them with exosomes derived from the *T. asiatica* adult worm (Tas-exo) on LoVo by RNA-seq analysis. Our results identified 348 genes as being significantly differentially expressed for the Tas-exo group when comparing with that of the NC group. Some of these genes are related to modulation of cell proliferation and cell autophagy. Surprisingly, autophagy and cell proliferation have crucial roles in the defense against parasites; accordingly, we detected cell proliferation and autophagy in LoVo cells by CCK8, immunofluorescence, and Western blotting, demonstrating that Tas-exo could inhibit LoVo cell proliferation and autophagy via AMPK pathway. When P62 and p-mTOR/mTOR expression were significantly increased, BeclinI and pAMPK/AMPK were significantly decreased. These results expand our understanding of parasite-host interactions mediated by exosomes.

## 1. Introduction

*Taenia asiatica* is a zoonotic parasitic flatworm that has been a major burden on public health systems and food safety for many years. Its larvae mainly infect swine liver, contributing to cysticercosis; adult parasites infect the human intestine, causing human taeniasis [1]. Due to the consumption of raw or undercooked pig liver, *T. asiatica* transmission has an important ethnogeographical association. Distribution is primarily in Taiwan, South Korea, Indonesia, the Philippines, Thailand, southwestern China, Vietnam, Japan, and Nepal [2,3,4]. Whether *T. asiatica* can cause human cysticercosis is not yet clarified; if so, then *T. asiatica* infection could be one of the most neglected tropical diseases [5]. *T. asiatica* evolved sophisticated mechanisms that are beneficial during the infection process and for long-term survival in the human intestine; however, there are no reports demonstrating the interaction between the host and tapeworms. Host-parasite interactions during infection may provide new insights into the sophisticated molecular mechanisms at work.

Parasites communicate with hosts by excretory-secretory pathways. As exosomes within the excretory-secretory products were recently identified as a mechanism for effectively mediating parasite-host interactions for host manipulation, it follows that the host would also use this pathway as a defense mechanism [6,7,8]. Exosomes are a type of ubiquitous, membrane-surrounded nanovesicle ranging from 30 to 100 nm. The multivesicular bodies (MVBs) form when the endosomal membrane fuses with lysosomes and the MVBs fuse with the plasma membrane, thus releasing an exosome [9,10]. Exosomes can efficiently deliver a huge amount of vital bioactive molecules, such as lipids, proteins, nucleic acids, and miRNAs to recipient cells [11,12]. Recent studies revealed that almost every type of cell, including tumor cells, bacteria, and parasites, abundantly release exosomes, which play an irreplaceable role under both normal and pathological conditions in regulating the immune response, cell proliferation, and inflammation in the host [13,14]. The increasing evidence demonstrates that helminths, including *Schistosoma japonicum* [15], *Fasciola hepatica* [16,17], and *Echinococcus granulosus* [18], also secrete exosomes into the environment that host cells can internalize. Most importantly, they can inhibit host immune responses by manipulating host gene expression to promote their survival and to achieve long-term parasitism [19]. The role of helminth-derived exosomes in parasite-host interactions is increasingly under investigation and their importance is frequently highlighted.

Recently, high-throughput RNA-sequencing (RNA-seq) technology, one of the latest transcriptomics methods, focused mainly on transcriptome analysis to accurately assess transcriptomic profiles by capturing many genes and identifying the precise sequence of nucleotide transcripts during host-parasite interactions [20,21]; this method is superior to traditional microarray analysis [21,22]. RNA-seq is a relatively accessible tool for elucidating transcriptomic changes in both hosts and parasites, which provides a full understanding of host defense mechanisms and expands our biological knowledge of these parasites and their interactions with hosts [20].

In a previous study, we focused on the descriptions of molecules packaged in the exosome [23]. However, the role of the interaction between adult *T. asiatica* worms and intestinal cells is not yet fully elucidated. In this study, high-throughput sequencing was used to analyze mRNA profiles of the LoVo cells treated with Tas-exo. The results not only indicated that various numbers of differentially expressed genes (DEGs) were involved in different biological processes, but also showed that exosomes could inhibit the proliferation and autophagy of LoVo cells, which may help *T. asiatica* defend against the host response and facilitate long-term survival in the intestine.

## 2. Materials and Methods

### 2.1. Cell Culture and Sample Treatment

Prepared exosomes derived from adult *T. asiatica* worms were preserved at −80 °C [23]. Colorectal cancer cell lines (LoVo) were generously provided by the Lanzhou Army General Hospital and cultured using RPMI-1640 medium (Hyclone, Logan, UT, USA) supplemented with 10% FBS (Gibco, Carlsbad, CA, USA), 100 U/mL penicillin (Hyclone), and 100 U/mL streptomycin (Hyclone) according to standard protocol. LoVo cells were seeded in 6-well plates (Beyotime, Shanghai, China) (5 × 10^4^ cells per well) and grown overnight in RPMI-1640 medium (Hyclone) with 10% exosome-free FBS (Gibco, Carlsbad, CA, USA) at 37 °C. Exosomes (28 µg/mL, 5 µL per well) were added to cells. Cells without treatment served as the control group. Each group was set to three biological replicates. The cells were cultured at 37 °C in 5% CO_2_ for 24 h, and collected and preserved at −80 °C.

### 2.2. RNA Extraction

The treated cells were collected by centrifugation and resuspended in Trizol. Following the manufacturer’s instructions, after centrifugation, the supernatant was saved and RNA was extracted. RNA concentration was detected using a NanoPhotometer spectrophotometer (Life Technologies, Carlsbad, CA, USA) and integrity was measured through the Agilent 2100 bioanalyzer (Agilent Technologies, Santa Clara, CA, USA). High-quality RNA samples were used to construct the sequencing library.

### 2.3. Library Construction and Illumina Sequencing

RNA-seq transcriptome libraries were obtained using a NEBNext^®^ UltraTM RNA Library Prep Kit from Illumina (New England Biolabs, Ipswich, MA, USA) at Novogene Co., Ltd. (Tianjin, China). mRNA was enriched with polyA through magnetic oligo (dT) beads and randomly fragmented into small pieces using a fragmentation buffer. Following Illumina’s protocol, cDNA was synthesized, their ends repaired, and then polyA was added and connected with the Illumina-indexed adaptors. Subsequently, cDNA with 250-300 bp was selected for PCR amplification and purified using AMPure XP beads, which successfully contributed to the library construction. Lastly, the paired-end libraries were sequenced on an Illumina Hiseq platform and 150 bp paired-end reads were generated.

### 2.4. Processing of Raw Reads; Sequence Alignment to the Reference Genome and Gene Expression Level

The FastQ raw reads were trimmed for analysis of quality and reliability. To obtain clean reads, a filter was used to remove ploy-N-containing reads, low-quality reads, and reads adapters from the raw data. The high-quality, paired-end clean reads were aligned to the corresponding reference genome (http://apr2018.archive.ensembl.org/Homo_sapiens/Info/Annotation, 28 January 2021) using HISAT2 v2.0.5. FeatureCounts (1.5.0-p3) was used to calculate the mapping read number for each gene. The unmapped reads were predicted as novel genes using StringTie (1.3.3b) [24]. FPKM for each gene was calculated on the basis of the length, and the read number was calculated.

### 2.5. DEG Analysis

Raw counts were normalized to adjust sequencing depth, then the differential expression levels of two groups were analyzed through DESeq2 version 1.6.3. After differential expression analysis, the resulting *P* values were adjusted for multiple testing using the Benjamini-Hochberg and Hochberg procedures. The genes with *P* value < 0.05 and |Fold Change| (|FC|) ≥1.2 were considered significantly differentially expressed.

### 2.6. Functional Analysis of DEGs

To determine the principal functions of the DEGs, all DEGs were subjected to gene ontology (GO) annotation using the GO database (http://www.geneontology.org, 28 January 2021). GO analysis was conducted to provide meaningful annotation of genes and gene product properties according to the cellular component (CC), molecular function (MF), and biological process (BP) domains. Kyoto Encyclopedia of Genes and Genomes (KEGG) analyses were performed using clusterProfiler (3.4.4). DEGs were significantly enriched in GO terms and KEGG pathways with *P* ≤ 0.05.

The protein-protein interaction (PPI) networks among the DEGs were analyzed using the STRING PPI database (http://string-db.org/, 28 January 2021), which included direct and indirect associations between proteins. After analyzing the results from STRING analysis and expression change information for each DEG, the association network was created using Cytoscape_v3.6.1 software.

### 2.7. Real-Time qPCR (RT-qPCR) Validation

RT-qPCR was performed to further validate randomly selected DEGs. Total RNA was extracted using Trizol reagent (Invitrogen, Carlsbad, CA, USA) and cDNA was synthesized using PrimeScript™RT reagent Kit with gDNA Eraser (Perfect Real Time) (Takara, Japan) according to the manufacturer’s protocol. RT-qPCR was performed using an abm^®^EvaGreen qPCR MasterMix no dye kit (Takara, Japan) on an ABI7500 thermocycler (Thermo Fisher Scientific, Waltham, MA, USA) using the following steps: initial activation at 95 °C for 10 min, followed by 40 cycles of 94 °C for 15 s, and 60 °C for 1 min. Each assay was performed in triplicate, and GAPDH was used as an internal control gene. The primers used are shown in supplementary Table S1. Data from triplicate-independent experiments were evaluated using online software. Relative DEG abundance was calculated using the 2^−ΔΔCt^ method.

### 2.8. LoVo Cell Viability Assay

The effect of exosomes on LoVo cell viability was measured using Cell Counting Kit (CCK-8) (Beyotime, Shanghai, China) according to manufacturer’s instructions. The LoVo cells were plated in 96-well plates at a density of 1 × 10^6^ cells/well and incubated for 24 h. Cells were then incubated with exosomes at concentrations of 100, 150, and 200 µg/mL in 100 μL complete RPMI 1640 medium with 5% FBS exosome-free or complete RPMI 1640 medium. After incubating for 48 h, 10 μL of CCK-8 solution was added to each well and continued to incubate for another 4 h. Absorbance values were then measured at 450 nm. Data from triplicate-independent experiments were evaluated. The data of every group were statistically analyzed by *t*-test (mean ± SD, * *P* < 0.05, ** *P* < 0.01, *** *P* < 0.001, and **** *P* < 0.0001).

### 2.9. Autophagy Detection

#### 2.9.1. Sample Treatment

LoVo cells were seeded in 6-well plates (5 × 10^4^ cells per well) and grown overnight in RPMI-1640 medium (Hyclone) with 10% exosome-free FBS (Gibco) at 37 °C. Exosome (28 µg/mL, 5 µL per well) and chloroquine (CQ) (200 µM, 2 µL per well) as a positive control and dimethyl sulfoxide (DMSO) diluted in PBS (1:1) as a solvent control were added to the cells. Cells without any treatments served as the control group. Each group was plated as three biological replicates. The cells were cultured at 37 °C in 5% CO_2_ for 72 h, after which the treated cells were collected. The total proteins were extracted by protein lysate (Beyotime, China) and preserved at −80 °C.

#### 2.9.2. Fluorescence Observation

LoVo cells were seeded in 6-well plates (5 × 10^4^ cells per well) and grown overnight in RPMI-1640 medium (Hyclone) with 10% exosome-free FBS (Gibco) at 37 °C. Both Adenovirus Expressing mCherry-GFP-LC3B fusion protein (Ad-mCherry-GFP-LC3B) and Adenovirus Expressing mCherry-GFP-LC3B fusion protein (Ad-mCherry-GFP-LC3B) (Beyotime, China) served as detectors for the autophagy phenomenon. The experiment was divided into the following 5 groups: (1) 10 μg exosome + 30μL Ad-mCherry-GFP-LC3B (MOI = 10); (2) 30 μL Ad-mCherry- GFP-LC3B (MOI = 10); (3) 10 μg exosome + 30 μL Ad-mCherry-P62 (MOI = 10); (4) 30 μL Ad-mCherry-P62 (MOI = 10), and (5) untreated cells. Next, cells were cultured at 37 °C in 5% CO_2_ for another 72 h. The treated cells were then observed under a laser scanning confocal microscope (LSCM) at 75 kv.

#### 2.9.3. Western Blot

The total proteins were separated by 15% and 10% SDS-PAGE and transferred onto PVDF membranes. The membranes were blocked with 5% skim milk at 4 °C overnight. The membranes were probed with different primary antibodies, rabbit anti-BeclinI antibody (1:2000), rabbit anti-P62/SQSTM1(P62) antibody (1:1000), rabbit anti-AMPK (1:2000) and anti-p-AMPK antibodies (1:2000), mouse anti-mTOR (1:2000), and anti-p-mTOR (1:2000) for 1 h with shaking at room temperature. After three PBST washes, the membranes were incubated with mouse anti-β-actin antibody (1:2000), and goat antirabbit IgG/HRP and goat anti-mouse IgG/HRP (1:2000) were added. All antibodies were purchased from Abcam (Burlingame, CA, USA). The membranes were incubated with ECL-luminous fluid (Beyotime, China), and mixed with solutions A and B. The membranes were scanned, and a high-resolution image was exported and analyzed by Image J. Data were analyzed by GraphPad Prism 6.0. Data from each group were analyzed by *t*-test (mean ± SD, * *P* < 0.05, ** *P* < 0.01, and *** *P* < 0.001). All experiments were repeated three times.

## 3. Results

### 3.1. Transcriptome Quantification Analysis

A total of 92,723,852 raw reads were obtained from the RNA library, including the NC (46,317,162)- and Tas-exo (46,406,690)-treated groups (https://www.ncbi.nlm.nih.gov/sra/PRJNA682788, 30 January 2021). After removing low-quality reads, 89,365,975 clean reads (NC: 44,418,486; Tas-exo: 44,947,489) were obtained (as illustrated in Table 1). Clean reads were queried against the latest reference genome (http://apr2018.archive.ensembl.org/Homosapiens/Info/Annotation, 30 January 2021). For the NC and Tas-exo samples, 42,321,430 and 42,695,406 reads were mapped to the reference genome with mapping rates of 95.28% and 94.99%, respectively.

### 3.2. DEGs Analysis

**Figure 1:**
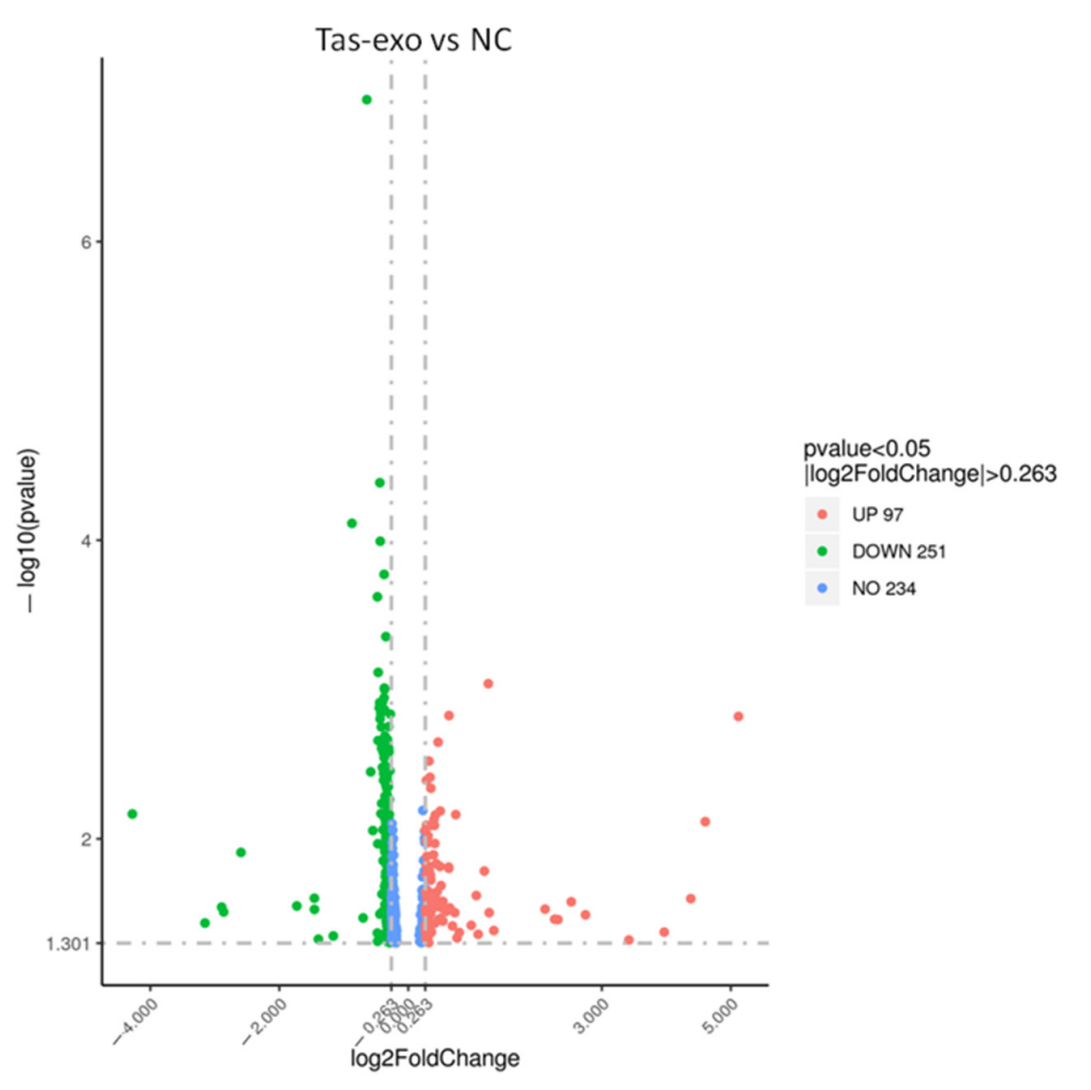
Volcano plot of global differentially expressed genes (DEGs). Red dots (up), significantly upregulated genes (*P* < 0.05, fold change > 1.2); green dots (down), significantly downregulated genes (*P* < 0.05, fold change > 1.2).

**Figure 2:**
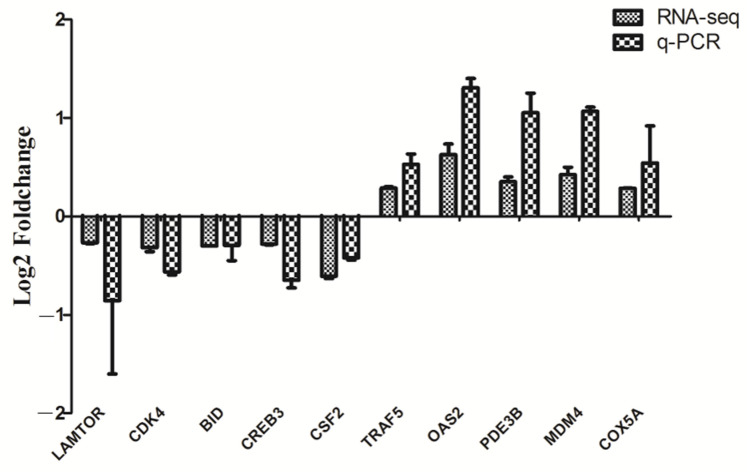
RT-qPCR validation on 10 randomly selected DEGs. Ten genes from RNA-seq data were randomly selected and confirmed by RT-qPCR. mRNA levels of Tas-exo and NC groups obtained from RNA-seq analysis were compared with those of RT-qPCR results. These two results were correlated using a form of log2Foldchange. Data from triplicate-independent experiments were evaluated using online software. Relative DEG abundance was calculated using 2^−ΔΔCt^ method.

A total of 348 genes were identified as significantly differentially expressed in Tas-exo group compared with those of the NC group (|FC| ≥ 1.2 and *P* < 0.05). Among the 348 genes, 97 were upregulated and 251 were downregulated (as illustrated in Additional file 1, Figure 1). Ten DEGs, including late endosomal/lysosomal adaptor and MAPK and mTOR activator (LAMTOR), cyclin dependent kinase 4 (CDK4), TNF receptor-associated factor 5 (TRAF5), 2′-5′-oligoadenylate synthetase 2 (OAS2), phosphodiesterase 3B (PDE3B), mouse double minute 4 (MDM4), BH3-interacting domain death agonist (BID), cAMP response element binding protein 3 (CREB3), cytochrome c oxidase subunit 5A (COX5A), and colony-stimulating factor (CSF2) were chosen for qRT-PCR analysis. Results showed that all genes showed a similar expression pattern, which was observed in the integrating analysis (as illustrated in Figure 2). LAMTOR, CDK4, TRAF5, BID, and CSF2 were downregulated compared with in the NC group, while OAS2, PDE3B, MDM4, CREB3, and COX5A were upregulated. These results confirmed that the DEGs identified by RNA-seq were relatively reliable.

### 3.3. Functional and PPI Analysis of DEGs Were Identified

**Figure 3:**
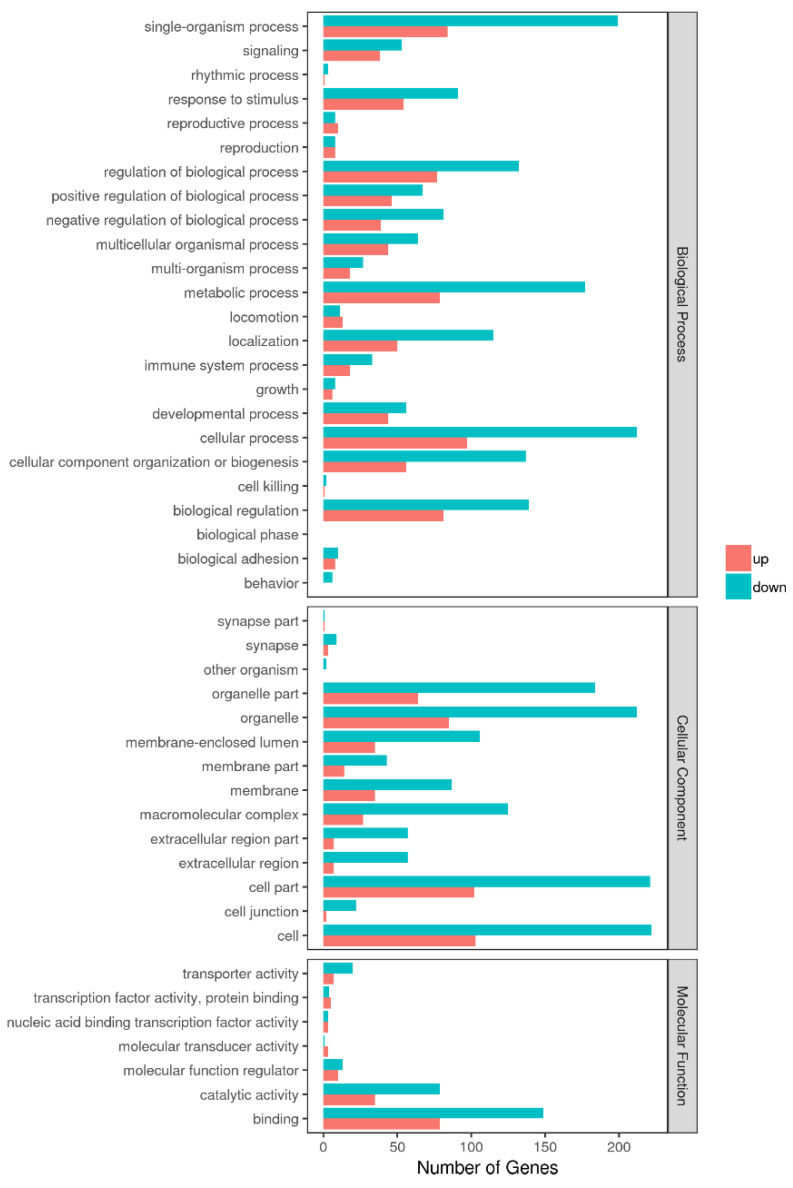
GO enrichment analysis of DEGs in Tas-exo vs NC; GO terms (X axis). Enrichment ratio of genes shown as GO terms for biological process (BP), cellular component (CC), and molecular function (MF).

**Figure 4:**
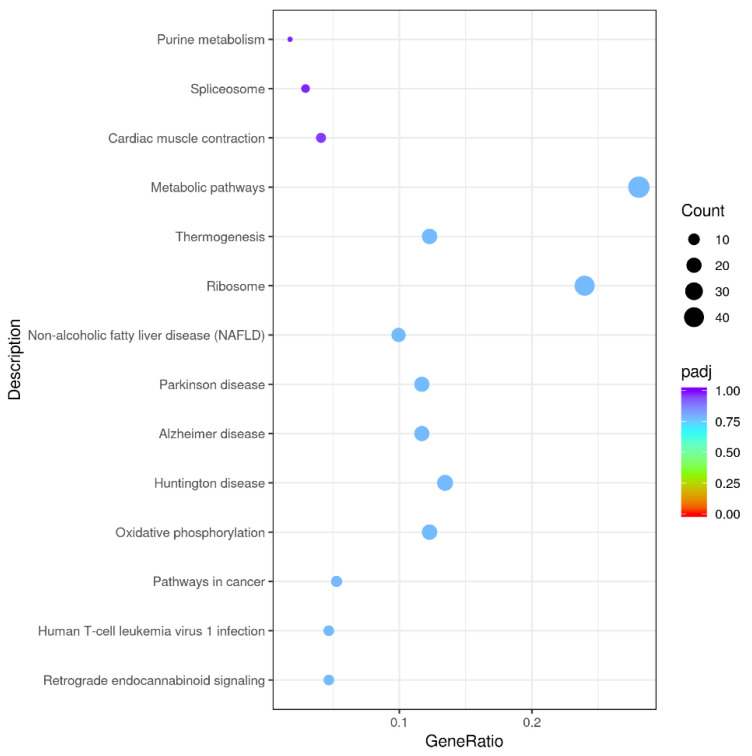
Kyoto Encyclopedia of Genes and Genomes (KEGG) functional annotations of DEGs. Enrichment of DEGs in different signal pathways. X axis represents GeneRatio, which contains amount of DEGs enriched in pathway; Y axis represents pathway (*P* adjusted < 0.05).

Among 348 DEGs, the upregulated and downregulated DEGs were annotated to 42 and 44 GO terms, respectively. The most annotated GO terms were cell, cell part, organelle, single-organism process in CC (cellular component), cellular process, metabolic processes, biological regulation, localization in BP (biological process), and binding in MF (molecular function; as illustrated in Figure 3). These DEGs were enriched in different KEGG pathways, including the metabolic and oxidative-phosphorylation pathways, and the ribosome. The top 14 pathways are shown in Additional File 2 and Figure 4.

**Figure 5:**
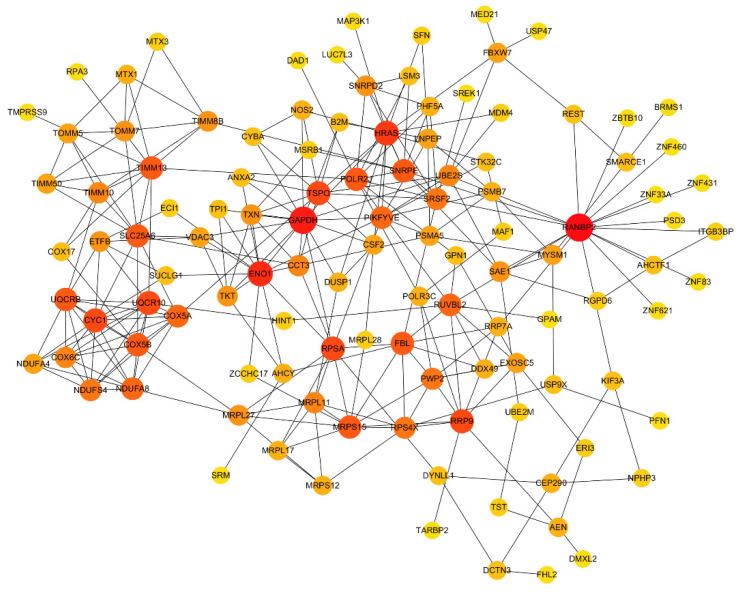
Protein-protein interaction (PPI) network analysis of DEGs in Tas-exo vs NC groups. PPI network constructed using selected DEGs from database. Node size and color shade represent number of interacting genes; node represents genes.

DEGs were queried for interactions using the STRING database, and 1530 interaction relationships were obtained. To intuitively clarify some relations between proteins, some genes were selected for PPI network analysis through Cytoscape_v3.6.1 (as illustrated in Figure 5). In this network, the translocase of the inner mitochondrial membrane (TIMMB), the ran-binding protein 2 (RANBP2), and the translocator protein (TSPO) interacted with 10, 17, and 11 proteins, respectively.

### 3.4. Exosomes Inhibit LoVo Cell Proliferation

**Figure 6:**
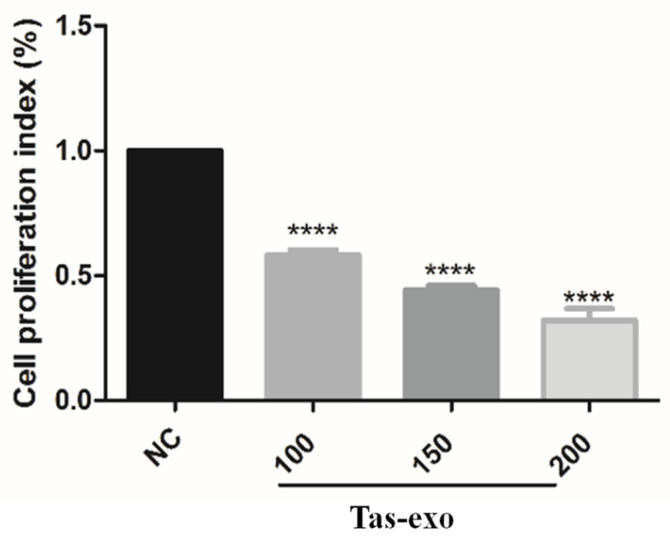
Effect of *T. asiatica*-derived exosomes at different concentrations on LoVo cell proliferation. Cell proliferation in Tas-exo group and NC group evaluated by CCK-8 assay. Exosome concentrations consisted of 100, 150, and 200 μg/mL, respectively. Data presented as mean ± SEM (*n* = 3; **** *P* < 0.0001).

Cell-proliferation ability was measured using CCK-8 at different exosome concentrations of 100, 150, and 200 µg/mL, and results indicated that, with increasing dosage of exosome, the proliferation index of LoVo cells among these three concentration groups was significantly lower than that of the NC group after 24 h (**** *P* < 0.0001). This demonstrated that LoVo cell proliferation was inhibited by exosomes from adult *T.* *asiatica* worms, and that there was significant dose-dependent difference in cell-proliferation inhibition (as illustrated in Figure 6).

### 3.5. Exosomes Suppress Autophagy in Exosome-Treated LoVo Cells

**Figure 7:**
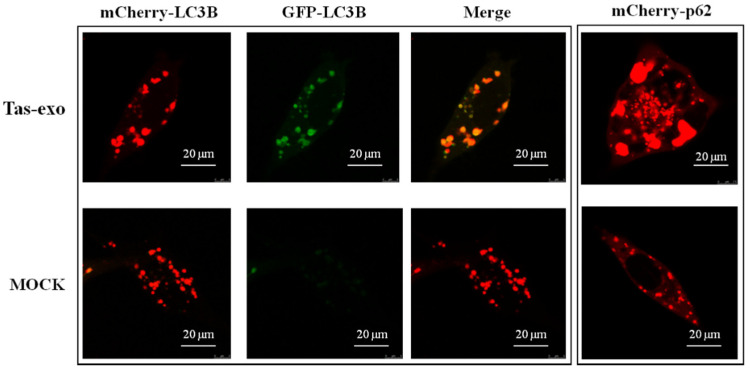
Fluorescent observation of exosome inhibition of LoVo cell autophagy. Fluorescence observed in exosome + Ad-mCherry-GFP-LC3B group and NC group, and exosome + Ad-mCherry-P62. These groups were observed using a laser scanning confocal microscope (LSCM) at 75 kv.

Autophagy is an indispensable part of the defense against parasites [25]. To detect the effect of exosomes on host autophagy, LoVo cells were transfected with Ad-mCherry-GFP-LC3B and Ad-mCherry-P62 plasmid (as illustrated in Figure 7). Results showed that red and green fluorescent signals could be observed together under the confocal microscope in the Ad-mCherry-GFP-LC3B group; when these two fluorescent signals were merged, yellow fluorescence emerged, indicating autophagy inhibition. The group of LoVo cells treated with exosomes and Ad-mCherry-P62 showed obvious red speckles, and the highly expressed P62 protein was significantly increased in aggregation, suggesting that the degradation of P62 proteins was inhibited by exosomes. In addition, the group of LoVo cells treated with Ad-mCherry-P62 displayed red puncta within the cytoplasm, which further suggests that exosomes could suppress autophagy in LoVo cells.

**Figure 8:**
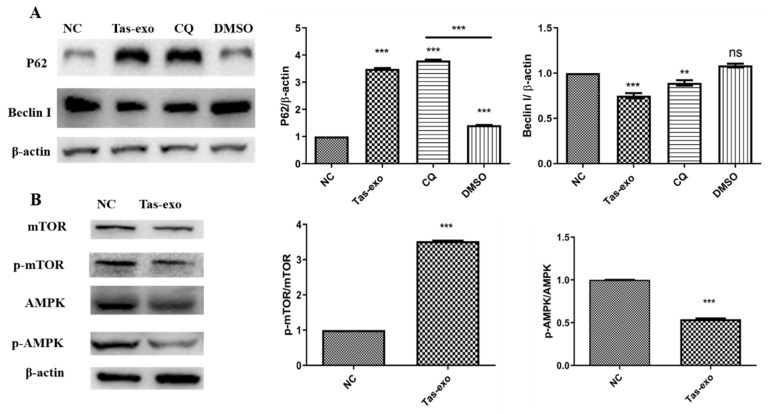
Western blot analysis of autophagy-related protein-expression changes and changes in autophagy-pathway-related proteins in LoVo cells after exosome treatment. (**A**) BeclinI and P62 levels evaluated in Tas-exo and NC groups. (**B**) AMPK and p-AMPK levels, and mTOR and p-mTOR levels evaluated in Tas-exo and NC groups. Analysis of variance performed with *t*-test; data shown as mean ± SD (** *P* < 0.01, and *** *P* < 0.001).

P62 and BeclinI are two markers of autophagy, and protein-level changes can be used to evaluate whether autophagy has occurred. In this study, CQ, as an autophagy inhibitor, was considered to be a positive control to evaluate the inhibition of autophagy. Our results showed that the expression of P62 was significantly increased and BeclinI was significantly decreased (*P* < 0.05) in the Tas-exo group in comparison with that of the NC and CQ groups (as illustrated in Figure 8A), indicating that Tas-exo could significantly inhibit LoVo cell autophagy after 72 h. Moreover, further investigation of the autophagy pathway showed that p-mTOR/mTOR (*P* < 0.0001) were significantly upregulated, and pAMPK/AMPK (*P* < 0.01) were significantly downregulated in the Tas-exo group when compared to that of the NC group (as illustrated in Figure 8B), indicating that the phosphorylation level of AMPK was decreased. This further revealed that Tas-exo may inhibit LoVo cell autophagy through the AMPK pathway.

## 4. Discussion

Exosomes secreted by parasites are considered to be “bridges”, allowing for parasites to communicate with their host during infection; they act as another mechanism through which helminths manipulate their hosts [26]. Normally, transcriptome analysis was reported to assess pathogen gene expression against the host, which provided the opportunity to better understand the pathogen infection mechanism [27]. Our previous study identified only *T. asiatica*-derived exosomes, and described molecules packaged in the exosome. To further explore the role of *T. asiatica*-derived exosomes in the interaction process between worms and intestinal cells, this study first identified the expression level of DEGs in LoVo cells treated by Tas-exo. Hundreds of genes were significantly modulated by Tas-exo. Additionally, cell autophagy was remarkably inhibited, implying that Tas-exo plays a key role in mediating the interactions between *T. asiatica* and host cells.

Since microarray and RNA-seq analysis can simultaneously detect the expression levels of thousands of genes in the parasite and human genomes, it is widely used to explore a huge amount of biodata on parasite-host interactions. There were many studies investigating transcriptome change in parasitic infection (*Toxoplasma gondii*, *Cryptosporidium,* etc.) using RNA-seq analysis [24,28], but mRNA changes in host cells treated with helminth-derived exosome remain unclear, especially in Taeniidae. Most importantly, exosomes are closely linked to the association between *T. asiatica* and host. Therefore, our study focused mainly on transcriptome change analysis of LoVo cells stimulated with *T. asiatica-*derived exosomes. We identified 251 downregulated genes in LoVo cells treated with Tas-exo, which showed that 72% of DEGs were significantly inhibited by exosomes. A possible explanation is that tapeworms create a beneficial microenvironment for parasitism. A recent study showed that EVs similarly secreted by *Schistosomiasis mansoni* cocultured with human umbilical vein endothelial cells (HUVECs) led to the production of DEGs associated with vascular endothelial contraction, coagulation, arachidonic acid metabolism, etc. [29], which implied that helminth-derived EVs could be involved in intravascular parasitism. Moreover, different kinds of helminth-derived vesicles may have different effects on host cells due to the different positions in which they parasitize [29]. Consequently, the downregulated expression of 251 genes induced by Tas-exo may contribute to worm survival, suggesting that exosomes play a pivotal role in host–parasite interaction.

In our transcriptome analysis, qRT-PCR results showed that both BID and CDK4 were downregulated, and LoVo cell proliferation was significantly inhibited, suggesting that these two key genes might be related to host cell apoptosis and cell-proliferation regulation. BID, one of the proapoptotic proteins of the Bcl-2 family, could be involved in extrinsic apoptotic signaling between death receptors and mitochondria [30,31]. The activation of BID is required for inducing different types of cell apoptosis [32,33]; it is vital for viability in many cell types and reduces cell proliferation. CDK4 is a member of CDK4 cyclin-dependent kinases and a key regulator of cellular life [34]. It is widely reported to efficiently inhibit growth and proliferation in various cell types [35,36]. The above show that BID and CDK4 are both related to modulation of cell apoptosis and cell proliferation. Therefore, further study is required to determine which *T.asiatica-*derived exosome molecules target BID or CDK4 to regulate the relevant signaling pathway. Moreover, increasing evidence suggests that helminths can increase intestinal mucosa epithelium permeability, and that helminth-derived excretory products may influence the structure of the tight junction [37]. Our results, which showed that tapeworm-derived exosomes inhibited host cell proliferation, strongly support the notion that helminth excretions can modulate the surrounding tissue environment.

As a housekeeping process, autophagy plays an important role in maintaining the metabolic balance of eukaryotes via autophagosomes and lysosomes on infections and other physiological or pathological conditions [38]. The autophagy pathway plays a crucial role in resistance to bacterial, viral, and protozoan infection in metazoan organisms [39]. Autophagy also plays a significant role in apoptotic corpse clearance in the protozoan parasite lifecycle [40]. LAMTOR is known to regulate the mTOR signaling pathway, extracellular signal-regulated kinase activation on the late endosome, and endosomal biogenesis, which in turn modulates cell growth and proliferation, migration, and spreading [41,42]. A previous study demonstrated that *T. gondii* can activate host cell signaling, which regulates the autophagy process to evade clearance [43]. Moreover, P62 plays a significant role in different physical processes, such as cell proliferation, survival, and death, and it is an autophagy marker. It serves as a signaling hub regulating various signaling pathways, including mTOR [44,45]. Our results showed that LAMTOR expression was downregulated, clearly implying that Tas-exo could inhibit cell autophagy. The P62 proteins were significantly degraded, and pAMPK/AMPK was downregulated by Tas-exo, further indicating the inhibition of autophagy in LoVo cells through the AMPK pathway. Results provided evidence that *T. asiatica* could evade host attack and expulsion for long-term survival in human intestines by regulating normal host-cell functions, which expands our knowledge about parasite-host interaction mediated by exosomes, and provides information on the potential intrusion and escape mechanisms involved in tapeworm parasitism.

## 5. Conclusions

In our study, the expression profiles of mRNA were investigated, and a total of 348 genes were significantly differentially expressed and involved in different biological processes in LoVo cells incubated with Tas-exo. Results further revealed that exosomes could inhibit LoVo cell proliferation and autophagy, providing novel insights into the interactions mediated by exosomes between parasite and host.

## Figures and Tables

**Table 1:** Statistics of mRNA sequences of the mRNA libraries

Sample	Raw Reads	Low Quality	Clean Reads	Aligned Reads	Aligned (%)
NC_1	47345502	1831684	45513818	20,968,264	94.64
NC_2	47345502	1769238	43468294	18,910,028	94.98
NC_3	46368454	2095106	44273348	16,335,233	95.26
Tas_exo1	46105212	1542694	44562518	27,372,901	94.7
Tas_exo2	46002696	1340400	44662296	19,070,337	95.47
Tas_exo3	47112162	1494510	45617652	21,502,677	95.52

## Supplementary Materials

The following are available online at https://www.mdpi.com/article/10.3390/microorganisms9050994/s1, Table S1: The specific primers, Additional file 1: the data of Tas-exo vs NC, Additional file 2: the KEGG data of Tas-exo vs NC, Additional file 3: the data of PPI network.
