# Supplementary material for: Transcriptome Analysis and Autophagy Investigation of LoVo Cells Stimulated with Exosomes Derived from T. asiatica Adult Worms
Source: Microorganisms. 2021 May 5;9(5):994. doi: 10.3390/microorganisms9050994 (PMC8147967; doi:10.3390/microorganisms9050994)
Supplement: Supplementary file 1 [file microorganisms-09-00994-s001.zip › Table S1.pdf]

Table S1 The specific primers

Name of Primers	Sequences (5' to 3')
LAMTOR-F	CCCATCCCGTTCTCTGATTT
LAMTOR-R	CTGTACAACCAGCTCCTCTTT
CDK4-F	CTGCAGGTCATACCATCCTAAC
CDK4-R	TCGAGAGGTAGCCATTCTCA
BID-F	CCTTGCTCCGTGATGTCTTT
BID-R	CCGTTCAGTCCATCCCATTT
TRAF5-F	GCTTTGTGGCTCATTCTGTTT
TRAF5-R	AGAGATCCTCCAGGTCAGTT
CREB3-F	TCTAGAGGTTCCGGTAACTAGG
CREB3-R	AATCTTCAGGCTCACTCTCTTG
COX5A-F	GCTCGCTGGGTAACATACTT
COX5A-R	GGGCTCTGGAACCATATCATAG
CSF2-F	GTCTCCTGAACCTGAGTAGAGA
CSF2-R	GCTCCTGGAGGTCAAACATT
OAS2-F	CTGAGTTAGAGCCCAGGATTTC
OAS2-R	GATGTCTGCCTCATCCTCTTAC
MDM4-F	GTCACTTTAGCCACTGCTACT
MDM4-R	CACTTTGCTTCAGTTGGTCTTG
PDE3B-F	AGTTATGGCTGCCTGTCTTC
PDE3B-R	TCCTCCCTGGGTGATCATAA
GAPDH-F	CCCTTCATTGACCTCAACTACA
GAPDH-R	ATGACAAGCTTCCCGTTCTC
